# The Assessment of Geriatric Depression Among Primary Healthcare Physicians in Buraidah City, Saudi Arabia

**DOI:** 10.7759/cureus.62239

**Published:** 2024-06-12

**Authors:** Jarallah A Aljarallah, Chandra Sekhar

**Affiliations:** 1 Family Medicine, Family Medicine Academy, Qassim Health Cluster, Buraidah, SAU

**Keywords:** saudi arabia, buraidah, management of depression, knowledge, primary health care centres, physicians

## Abstract

Background: The global geriatric population is increasing, leading to a higher prevalence of non-communicable diseases, including depression. This condition often remains underdiagnosed and untreated disease.

Methodology: A cross-sectional study was conducted among 130 primary healthcare physicians (PCPs) in Buraidah to assess their practices in diagnosing geriatric depression from March 2023 to March 2024. After ethical committee approval, data were collected through a self-administered questionnaire, entered, cleaned, and analyzed with Statistical Package for the Social Sciences (IBM SPSS Statistics for Windows, IBM Corp., Version 21.0, Armonk, NY). Informed consent was obtained and the confidentiality of the participant information was maintained. Statistical tests, including the Chi-square test, were used for inference.

Results: Out of 130 PCPs, 85.4% (n=111) were diagnosing depression during their clinical practice. The most common depression scale used in their regular practice was the patient health questionnaire (PHQ)-2 (70%, n=91), followed by the Geriatric Depression Scale (53.8%, n=70). Nearly 26.2% (n=34) of physicians responded that there is no need for routine lab tests for the diagnosis of depression. Concerning the initial plan of depression management, nearly 76.2% (n=99) of physicians preferred non-pharmacological treatment. Regarding barriers to diagnosis of depression in elderly patients, nearly 76.2% (n=99) mentioned the need for more training about geriatric depression, followed by 70% for both short consultation time and the need for Ministry of Health guidelines. There was a statistically significant association observed between <35 years of age group and a preference for a high percentage of pharmacological therapy (P<0.05).

Conclusions: Based on the findings of the study, there was a good number (85.4%) of PCPs diagnosing depression in their clinics, and also three-fourths of the physicians (76.2%) preferred non-pharmacological treatment. Only one-fourth (26.2%) of the PCPs mentioned no lab is required for the diagnosis of depression.

## Introduction

One of the most often diagnosed mental illnesses in adults (18 years and above) is depression [1] and it is a common cause of disability all over the world [2]. Also, major depressive disorder (MDD) is a disabling disease that is characterized by symptoms such as interrupted sleep or hunger as well as other symptoms like depressed mood, reduced interests, cognitive decline, and difficulty with concentration. One in six persons will experience MDD at some point in their lifetime, with women experiencing it nearly twice as frequently as men. MDD has a complex etiology with a heritability of about 37%, according to estimates mentioned by Belmaker et al. Environmental variables such as childhood sexual, physical, or emotional abuse are very closely linked to the likelihood of having MDD. All facts of the disease cannot be explained by any known mechanism [3].

The global old age population is increasing, with notable trends observed in both developed and developing countries. The percentage of elderly individuals worldwide is predicted to nearly double from 12% to 22% between 2015 and 2050 (WHO, 2022). It is anticipated that the number of older individuals will rise from 900 million to 2 billion in absolute terms. We must understand the unique physical and mental health issues older persons experience. The WHO defines the elderly as those who are 60 years of age and older. Among older individuals, approximately 20% experience mental illness. Depression stands out as the most common mental health disorder in this age group, often coinciding with increased morbidity and mortality [4]. Late-life depression is associated with physical limitations, greater functional impairment, elevated healthcare expenses, higher suicide rates, and overall mortality [5]. Around 69% of depressed patients suffer from nonspecific somatic symptoms, which include back pain, neck pain, tiredness, headaches, and constipation [6].

Despite the high prevalence of this disorder, numerous studies have indicated that both the management and diagnosis rates among primary care patients remain inadequate [7]. Based on a study, primary care physicians correctly identified severe depression in only 34.9% of cases and recognized other milder forms of depression in 27.9% of patients [8]. The Kingdom of Saudi Arabia, one of the largest nations in the Middle East, is experiencing an increase in its geriatric population. It is projected that this population will reach nearly 2.5 million individuals, constituting approximately 7% of the total population by 2020 [9].

Elderly individuals face several challenges, often dealing with chronic illnesses such as depression, limited income, and reduced mobility. As a result, they tend to seek care at primary healthcare centers (PHCCs). The concept of depression being the “tip of the iceberg” highlights that visible symptoms represent only a fraction of the complex factors contributing to an individual’s mental health. By understanding these underlying factors, healthcare providers can tailor treatment approaches effectively [5]. Therefore, improving primary healthcare physicians’ (PCPs’) diagnostic capacity and knowledge, reducing stigma, and providing access to evidence-based treatment are critically necessary to increase the quality of life and medical results measures in the elderly population [10]. In a cross-sectional study conducted in the Riyadh region, 180 non-psychiatrist physicians were assessed for their knowledge and attitudes regarding psychiatric disorders, including depression and anxiety. The findings revealed that family practitioners exhibited a positive attitude toward psychiatric patients, while general practitioners and specialists displayed a negative attitude [11].

Another study was conducted among primary care physicians in the state of Michigan, USA, and 60% responded. The study showed positive attitudes regarding their abilities to identify and treat geriatric depression, but only 25% of the respondents used a screening tool regularly. The practice guidelines for treating depression were unknown to 41% of all doctors. Family doctors had higher levels of treatment confidence than general internists (85% vs. 50%) [12]. A study conducted in rural Illinois, USA, among 162 PCPs revealed the result that doctors' attitudes toward their role in treating elderly depressive patients were generally positive. Nonetheless, 45% of those who practice in rural areas reported a "gap" between the ideal and actual care [13].

Another study conducted among a random sample of 11% of the PCPs in Ontario, Canada, shows that many physicians limit their treatment to smaller doses (initial doses) and brief therapy sessions than what is necessary to achieve full clinical efficacy and avoid recurrence. This could be due to specialized literature and not being easily accessible to PCPs [14]. A study conducted in the Baghdad region of Iraq reported that 65.1% of participants were family doctors, and 65% achieved a high overall knowledge score. In summary, individuals who were family doctors, specialists, or older than 40 demonstrated significantly greater knowledge levels [15].

Building upon the context provided, the present study's objectives are to assess the knowledge, clinical practices, and barriers related to geriatric depression diagnosis and treatment among PCPs in Buraidah City, Saudi Arabia.

## Materials and methods

Study setting and target population

This study was carried out at the PHCCs in Buraidah City. The focus of the study was on physicians working at the selected PHCCs in the same city.

Study design

A cross-sectional study was conducted among PCPs through a combination method of data collection by electronic survey and hard copy distribution to physicians through a self-administered questionnaire.

Data collection

In order to create the questionnaire, we conducted a thorough review of PubMed and Google Scholar search engines to align with our research concept. Subsequently, we identified over 10 relevant studies related to our research topic. We built the questionnaire based on our objectives and some research gaps in the previous studies.

Objectives

The first one is to determine the demographic factors and their association with PHCC physicians’ knowledge about depression among elderly patients. The second objective is to find the primary care physicians' management and barriers to their perceptions of detecting and managing depression among elderly patients in Buraidah City.

Some of the new variables like taking a history from the patient before diagnosing depression, class of medication, common medicine prescription, and non-pharmacological intervention to manage the depression for their patients were included as variables in our study.

The questionnaire was developed based on previous studies conducted in Saudi Arabia [6], Bahrain [7], and the United States of America [13]. The US study provided open access to their questionnaire. We obtained approval from the corresponding authors of the Saudi Arabia and Bahrain studies to use specific variables and cited them appropriately. The questionnaire, including newly added variables, was validated by a psychiatric consultant and experienced research faculty at the Qassim Family Medicine Academy.

Our questionnaire includes mainly three parts (the first part is demographic data, and the second part is physicians’ knowledge of geriatric depression screening and diagnosis. The third part deals with the management and barriers aspects of PHCC physicians).

In our questionnaire, the first part included demographic characteristics like age, gender, duration of experience, and position of physicians. The second part denoted the patient history for diagnosis of depression, common symptoms presentation, type of depression scale, lab investigations before diagnosis, and same investigations after diagnosis before initiation of depression treatment. The third part of the questionnaire included the type of common medication used for depression, time of referral for the patients to a psychiatrist, follow-up of medication, and difficulties of diagnosis among geriatric patients. Initial treatment plans for geriatric management are classified as pharmacological and non-pharmacological treatment. Some of the categorical variables such as pharmacological treatment classified as selective serotonin uptake inhibitors (SSRIs), serotonin-norepinephrine reuptake inhibitors (SNRIs), and tricyclic antidepressants. Types of non-pharmacological treatment include exercise, cognitive-behavioral therapy (CBT), and interpersonal psychotherapy.

Sampling

As per Ministry of Health (MOH) statistics, there are 155 PHCCs working in Buraidah City; approximately 41 PHCCs are functional. About 170 PCPs are available from 41 PHCC and 60 family medicine residents in Buraidah City, Qassim Health Cluster. According to the OpenEpi sample size calculator (Rollins School of Public Health at Emory University, Atlanta, USA), the required sample for the study is estimated based on the total number of PHCC physicians in Buraidah which is 170, and the number of FM residents at FMA, which is 60, and hence the total PHCC physicians population is 230 in Buraidah, assuming a prevalence of 50%, with 95% confidence level and a design effect of one. Based on the above parameters, the sample estimate was 145.

Sampling method

The PHCCs were selected using the simple random method through MS Excel (Microsoft® Corp., Redmond, WA, USA). Hence, 60% of the PHCCs (25 PHCCs) were selected randomly. After the selection of the PHCC, all the physicians working at PHC included in the study based on the convenience sampling method were applied as per the criteria and participant interest.

Inclusion and exclusion criteria

PCPs of both genders working in Buraidah city were included, while physicians who were not interested in the study and those working at private hospitals, polyclinics, and other government hospitals were excluded.

Pilot study

After ethical committee approval, a pilot study was conducted on 15 physicians. The pilot study was conducted to ensure a good presentation, order of the questions, and technical feasibility. After the pilot study, we did not edit any questions of our questionnaire. The pilot study sample was not included in the main study sample.

Ethical considerations

Prior to commencing data collection for the study, we obtained ethical committee approval number 607-44-16482 from the Reginal Ethics Committee in Qassim. Informed consent was obtained from each participant, and the study ensured that no harm was inflicted. Subsequently, permission was granted by the PHC director, and the questionnaire was distributed to the relevant PCPs. Confidentiality and privacy of individual information were rigorously maintained.

Statistical analysis

Our independent variables are age, gender (male and female), position of physicians, type of lab test, type of pharmacological and non-pharmacological treatment, common symptoms of depression, and different depression scales. Dependent variables are pharmacological and non-pharmacological therapy, barriers to diagnosis like the elderly not accepting questions, short consultation time, need for specific screening, and need for more training. The data underwent entry, cleaning, coding, and analysis using Statistical Package for the Social Sciences (IBM SPSS Statistics for Windows, IBM Corp., Version 21.0, Armonk, NY). Among the PHCC physicians, we assessed knowledge about geriatric depression by self-administered questions of geriatric depression diagnosis, screening, type of depression scale, and presenting symptoms of the patients. Their answers were considered as yes or no options. Practice assessment among the PHCC physicians included questions on choosing the lab test and initial plan of management for depression (pharmacological and non-pharmacological treatment), the type of non-pharmacological treatment, and the class of pharmacological treatment. Responses were collected as yes or no options. Regarding the assessment of the barrier, a 3-point Likert scale ranging from disagree, neutral, and agree was used.

We calculated means and standard deviations for the continuous variable of age in the study. The null hypothesis states that there is no association between PHCC physicians' demographic characteristics and the initial plan of geriatric depression treatment. The alternative hypothesis suggests that there is an association between PHCC physicians' demographic characteristics and the initial plan of geriatric depression treatment. The Chi-square test was applied to check the hypothesis that demographic characteristics and the initial plan of geriatric depression treatment were related. Additionally, a Chi-square test was applied to all categorical variables such as pharmacological treatment and non-pharmacological treatment with socio-demographic characteristics of age category, gender, and position of physicians. The level of statistical significance was set at a probability (P) value less than or equal to 0.05.

## Results

Among the 130 PHCC physicians in our study population, the mean age was 32.37 years with a standard deviation (SD) of 7.23. The response rate among participants was 83.8% (130 out of 155). During the study, physicians screened their patients for depression: 81 (62.3%) conducted screenings, 45 (34.6%) occasionally screened in their clinics, and four (3.1%) did not screen at all. The mean follow-up duration in the study population was 2.67 weeks, with an SD of ±1.42. Additionally, the mean years of experience were 5.90 (SD ± 6.0). Approximately 75% of physicians were below the age of 35, and the age range in our study spanned from 25 to 65 years. Notably, nearly 6 out of 10 physicians (60.8%) were male (see Table 1).

**Table 1:** Demographic characteristics of the physicians working at PHCC of Buraidah City PHCC: primary healthcare center; FM: Family Medicine

Demographic Variables	Number of Participants	Percentage
Age in years ± SD	32.37 ± 7.23	
Experience of physicians in years ± SD	5.90 ± 6.0	
Age Category		
≤35 years	100	76.9
>35 years	30	23.1
Male	79	60.8
Female	51	39.2
GP (General Physician) + Intern (2)	21	16.1
FM Resident	56	43.1
Registrar/Specialist	39	30.0
Consultant	14	10.8

According to Table 1 in our study, 43.1% (n=56) of participants were Family Medicine residents, while registrar and specialist positions accounted for 30% (n=39). Additionally, more than three-fourths of the physicians (76.9%) fell into the age group below 35 years.

Table 2 illustrates that among PHCC physicians, 85.4% (n=111) were actively diagnosing depression during their clinical practice. On average, these physicians saw two patients per month. The most commonly used depression scale in their regular practice was the Patient Health Questionnaire (PHQ)-2, employed by 70% (n=91) of the physicians. Following that, the Geriatric Depression Scale (GDS) was also frequently utilized, with 53.8% (n=70) of physicians using it.

**Table 2:** Depression diagnosis, screening, and type of depression scale used among the PHCC physicians PHCC: primary healthcare center

Variables	Yes (%)	No (%)
Depression diagnosis practice by physicians	111 (85.4)	19 (14.6)
The mean number of patients seen per month ± SD	1.7=2 ± 2	
Depression scale used in their practice	Yes (%)	No (%)
Beck Inventory Depression Scale	15 (11.5%)	115 (88.5%)
Mini-Mental State Exam	41 (31.5%)	89 (68.5%)
Patient Health Questionnaire (PHQ-2)	91 (70%)	39 (30%)
Zung Self-Rating Depression Scale	6 (4.6%)	124 (95.4%)
Geriatric Depression Scale	70 (53.8%)	60 (46.2%)
Centre for Epidemiologic Studies Depression Scale (CES-D)	16 (12.3%)	114 (87.7%)

According to Table 3, 94.6% (n=123) of PHCC physicians reported sad mood symptoms of patients, while 92.3% (n=120) indicated a loss of interest symptoms. These symptoms serve as triggers for physicians to consider screening patients for depression. However, persistent pain and regular job dysfunction symptoms were less common factors leading physicians to screen their patients for depression, with 44.6% (n=58) reporting these symptoms.

**Table 3:** Screening status of the physicians based on the presenting symptoms of the patients in the study population

Symptoms	Yes (%)	No (%)
Sad mood	123 (94.6%)	7 (5.4%)
Anxiety	90 (69.2%)	40 (30.8%)
Multiple worries	73 (56.2%)	57 (43.8%)
Loss of interest or pleasure	120 (92.3%)	10 (7.7%)
Sleep disturbance	104 (80%)	26 (20%)
Persistent pain (headache, abdominal pain)	58 (44.6%)	72 (55.4%)
Regular job dysfunction	58 (44.6%)	72 (55.4%)
Decreased social participation	86 (66.2%)	44 (33.8%)
Decreased energy	93 (71.5%)	37 (28.5%)
Weight change (increased/decreased)	74 (56.9%)	56 (43.1%)

In Table 4, it was observed that approximately 26.2% (n=34) of physicians indicated that routine lab tests are unnecessary for diagnosing depression. Regarding the initial management plan for depression, approximately 76.2% (n=99) of physicians favored non-pharmacological treatments. Among these, 70.8% (n=92) of physicians preferred CBT, followed by exercise (16.2%, n=21). In terms of prescribing antidepressant medications, 45.4% (n=59) of physicians occasionally prescribed them, while 32.3% (n=42) always did, and 22.3% (n=29) did not prescribe medication. The most commonly chosen class of pharmacological treatment by physicians was SSRIs, with escitalopram (46.9%, n=61) being the most frequently prescribed medication.

**Table 4:** Physicians' response about routinely ordered lab tests to diagnose and their management plans in the study population CBC: complete blood count; TSH: thyroid-stimulating hormone

Lab Test	Number	Percentage
CBC	3	2.3
Blood chemistry	2	1.5
TSH	19	14.6
No lab needed	34	26.2
More than one lab test indicated	72	55.4
Initial plan of treatment (n=130)	Pharmacological	Non-pharmacological
Response	31 (23.8%)	99 (76.2%)
Type of non-pharmacological treatment (n=130)	Number	Percentage
Exercise	21	16.2
Cognitive behavioural therapy	92	70.8
Interpersonal psychotherapy	12	9.2
None	5	3.8
Pharmacological treatment (n-130)	Number	Percentage
Yes	42	32.3
No	29	22.3
Sometimes	59	45.4
Class of pharmacological treatment (n=130)	Number	Percentage
Selective serotonin uptake inhibitors (SSRIs)	95	73.1
Serotonin-norepinephrine reuptake inhibitors (SNRIs)	3	2.3
Tricyclic antidepressants	3	2.3
None	29	22.3
Most frequent medication use (n=130)	Number	Percentage
Escitalopram	61	46.9
Amitriptyline	3	2.3
Citalopram	8	6.2
Fluoxetine	5	3.8
Sertralin	3	2.3
None	50	38.4

According to Table 5, the most prevalent symptoms leading to referrals to psychiatric hospital were suicidal risk (97.7%, n=127), psychotic symptoms (96.9%, n=126), harm to others (96.2%, n=125), and poor response to treatment (94.6%, n=123).

**Table 5:** Physicians' referral indications based on the study group's symptoms and signs

Symptoms and Signs Indicating the Need for Referral	Agree (%)	Disagree (%)
Had a poor response (persistent depressive symptoms) on treatment	123 (94.6%)	7 (5.4%)
Need for augmentation	84 (64.6%)	46 (35.4%)
Unmasking of hypomania or mania	120 (92.3%)	10 (7.7%)
Psychotic symptoms	126 (96.9%)	04 (3.1%)
Concerns regarding suicide risk	127 (97.7%)	03 (2.3%)
Harm to others	125 (96.2%)	05 (3.8%)
Intolerable side effects of medication	96 (73.8%)	34 (26.2%)
Multiple comorbidities	79 (60.8%)	51 (39.2%)
Neurocognitive disorder	117 (90%)	13 (10%)

According to Table 6, approximately 76.2% (n=99) of physicians expressed the need for additional training related to geriatric depression diagnosis. Additionally, 70% (n=91) of physicians cited the short consultation time as a factor. Furthermore, 70% (n=91) of physicians indicated that there are currently no MOH or Quality Health Care (QHC) guidelines specifically addressing the diagnosis and treatment of depression in the geriatric population.

**Table 6:** Physicians' opinions on the status of factors that make it difficult to diagnose depression in elderly patients

Factors That Make It Difficult to Diagnose Depression	Agree (%)	Neutral (%)	Disagree (%)
Elderly patients don't accept questions	76 (58.5%)	34 (26.2%)	20 (15.4%)
Short consultation time	91 (70%)	25 (19.2%)	14 (10.8%)
Need for a specific screening scale	60 (46.2%)	48 (26.9%)	22 (16.9%)
Need for more training about geriatric depression	99 (76.2%)	19 (14.6%)	12 (9.2%)
Guidelines	Yes (%)	No (%)	
Availability of Ministry of Health (MOH)/Qassim Health Cluster (QHC) guidelines for depression diagnosis and treatment	39 (30%)	91 (70%)	

Table 7 indicates that among physicians in the <35 years age group, 29% preferred pharmacological therapy. In contrast, physicians aged >35 years showed a preference for pharmacological therapy in only 6.7% of cases. A statistically significant association was observed between <35 years age group physicians and their preference for pharmacological therapy (P<0.05). However, no statistically significant association was found between the initial management of pharmacological therapy and gender or the position of the doctor (P>0.05).

**Table 7:** Demographic factors associated with the initial plan of geriatric depression treatment GP: General Physician; FM: Family Medicine

Variables	Pharmacological Therapy	Non-pharmacological Therapy	Total
≤35 years	29 (29.0%)	71 (71.0%)	100 (100.0%)
>35 years	2 (6.7%)	28 (93.3%)	30 (100.0%)
Chi-square (x^2^)=6.338, 1df, P=0.012; OR=5.71, CI: 1.27-25.58			
Males	20 (25.3%)	59 (74.7%)	79 (100.0%)
Females	11 (21.6%)	40 (78.4%)	51 (100.0%)
x^2^=0.240, 1df, P=0.624; OR=1.23, CI: 0.533-2.850			
GP	6 (28.6%)	15 (71.4%)	21 (100.0%)
FM Resident	12 (21.4%)	44 (78.6%	56 (100.0%)
Registrar/Specialist	10 (25.6%)	29 (74.4%)	39 (100.0%)
Consultant	3 (21.4%)	11 (78.6%)	14 (100.0%)
x^2^=0.553, 3df, P=0. 907			

Table 8 reveals that physicians under the age of 35 agreed that there is a need for specific screening for geriatric depression at a rate of 38%. Conversely, physicians aged 35 and older concurred at a higher rate of 73.3%. A statistically significant association was observed between the >35 years age group and agreed that need for a specific screening program for geriatric depression (P<0.05). However, no statistically significant association was found between gender and other barriers related to diagnosis and treatment aspects (such as elderly patients not accepting questions, short consultation time, need for specific screening, and the need for more training) within the study group (P>0.05).

**Table 8:** Different barriers of diagnosis and treatment aspects with socio-demographic factors in the study

Variables	Elderly Don’t Accept Questions			
Age	Agree	Neutral	Disagree	Total
≤35 years	60 (60.0%)	24 (24.0%)	16 (16.0%)	100 (100.0%)
>35 years	16 (53.3%)	10 (33.3%)	4 (13.3%)	30 (100.0%)
x^2^=1.051, 2df, P=0.591				
Variables	Short consultation time			
≤35 years	71 (71.0%)	18 (18.0%)	11 (11.0%)	100 (100.0%)
>35 years	20 (66.7%)	7 (23.3%)	3 (10.0%)	30 (100.0%)
x^2^=0.425, 2df, P=0.809				
Variables	Need for specific screening			
≤35 years	38 (38.0%)	44 (44.0%)	18 (18.0%)	100 (100.0%)
>35 years	22 (73.3%)	4 (13.3%)	4 (13.3%)	30 (100.0%)
x^2^ =12.417, 2df, P<0.05				
Variables	Need more training			
≤35 years	79 (79.0%)	15 (15.0%)	6 (6.0%)	100 (100.0%)
>35 years	20 (66.7%)	4 (13.3%)	6 (20.0%)	30 (100.0%)
x^2^ =5.405, 2df, P=0.067				
Variables	Elderly don’t accept questions			
Male	43 (54.4%)	20 (25.3%)	16 (20.3%)	79 (100.0%)
Female	33 (64.7%)	14 (27.5%)	4 (7.8%)	51 (100.0%)
x^2^ =3.716, 2df, P=0.156				
Variables	Short consultation time			
Male	56 (70.9%)	14 (17.7%)	9 (11.4%)	79 (100.0%)
Female	35 (68.6%)	11 (21.6%)	5 (9.8%)	51 (100.0%)
x^2^=0.334, 2df, P=0.846				
Variables	Need for specific screening			
Male	34 (43.0%)	31 (39.2%)	14 (17.7%)	79 (100.0%)
Female	26 (51.0%)	17 (33.3%)	8 (15.7%)	51 (100.0%)
x^2^=0.792, 2df, P=0.673				
Variables	Need more training			
Male	56 (70.9%)	15 (19.0%)	8 (10.1%)	79 (100.0%)
Female	43 (84.3%)	4 (7.8%)	4 (7.8%)	51 (100.0%)
x^2^=3.542, 2df, P=0.170				

## Discussion

The current cross-sectional study was conducted during the period from March 2023 to March 2024 with the objective of PHC physicians' perceptions, management, and barriers to detect depression among elderly patients in Buraidah City. Elderly patients have personal and demographic situations, lifestyle factors, economic factors, and other locomotor factors that play an important role in the maintenance of health and disease.

In the current study, about 62.3% of physicians screened patients for depression, 34.6% sometimes screened patients in their clinics, and 3.1% did not screen patients at all. The study conducted in the Asir region stated that nearly one-third of physicians do not use any screening procedure among geriatric attendees [6]. State of Michigan, USA revealed that only 25% of the respondents used a screening tool regularly among PHCC physicians [12]. Bahrain study revealed that 9.8% of physicians never screened for geriatric depression [7]. Less screening strategies were adopted in a study conducted in the USA in 1997 [16]. Another study emphasized that regular screening of the elderly at PHCC is essential for the early detection of depression [17]. In our study, approximately 85.4% of physicians diagnose depression during their clinical practice. A Bahrain study stated that 72.7% of physicians diagnose a depression condition among geriatric patients [7].

In our study, the most frequently used depression scale in regular clinical practice was the PHQ-2, with approximately 70% of physicians utilizing it. Following closely, the GDS was employed by 53.8% of participants. However, a systematic review conducted in Durham reported different findings. According to that study, the GDS was commonly used, and its sensitivity and specificity were both 82%. The review also highlighted the prevalence of other depression screening tools among older adults [18].

In our study, 94.6% of physicians recognized sad mood, while 92.3% identified loss of interest as a crucial symptom warranting depression screening. A study conducted in Asir yielded comparable results, with 97.9% noting loss of interest and 87% noting sad mood [6]. Similarly, studies in the USA emphasize low mood and loss of interest as predominant symptoms for diagnosing depression [19]. In a study conducted by Robinson et al., they emphasized that symptoms such as loss of interest and low mood play crucial roles in diagnosing depression among elderly individuals. These symptoms serve as concise indicators, and considering them is essential when assessing depression [20].

Nearly 26.2% of physicians responded that there is no need for routine lab tests for the diagnosis of depression in the present study. Only 14.6% of PHCC physicians prescribed thyroid-stimulating hormone (TSH) levels for elderly patients. In the Aseer study, about 35.8% of study participants did not prescribe any lab test for screening of depression, and also found that about a maximum of 54.5% of participants were ordered serum TSH level [6]. In a study conducted by Talaei et al., involving 174 hypothyroid patients who used the Beck Inventory scale for depression diagnosis, the receiver operating curve (ROC) was also applied. The analysis revealed that the cut-off point for TSH value was 2.5 MIU/L, achieving 89.66% sensitivity for depression detection. Additionally, a TSH value of 4 MIU/L was considered indicative of severe depression [21]. Other studies conducted in Brazil [22] as well as the American Thyroid Association also supported that there is a significant association between TSH level and the development of depression [23].

About 76.2% of physicians preferred non-pharmacological treatment for the initial plan of depression management. Of these, 70.8% of physicians preferred CBT, followed by exercise 16.2%. A study conducted by Dorow et al. [24] stated that the first choice of preference was non-pharmacological therapy for depression such as psychotherapy, exercise, and talking to friends and family, also observed a similar line of observation strength of 4.1 out of 5-point Likert scale for psychotherapy with Raue et al. [25].

A study conducted in the USA stated that psychotherapy is beneficial for treating geriatric depression along with various somatic treatments. Among these treatments, antidepressants are the most frequently used. Mild to moderate depression typically responds well to either medication or psychotherapy. When both treatment options are available in the patient’s community, it is reasonable for the patient to choose the approach that best aligns with their preferences and needs [26].

Among PHC physicians who prescribe antidepressant medications, 45.4% do so occasionally, while 32.3% always prescribe them. About 73.1% of physicians selected the class of pharmacological treatment as SSRIs. Of these, escitalopram was the most frequently prescribed medication by physicians, accounting for 46.9% of prescriptions. Supporting our study's findings, an Indian study revealed that SSRI prescriptions by physicians were common, accounting for about 79.2%. Escitalopram prescriptions comprise 40% and a small portion is occupied with tricyclic antidepressants [27]. Antidepressants continue to be the most frequently used somatic treatment. In primary care settings where psychotherapy is not accessible, antidepressants serve as the primary treatment. The best studies are recommended second-generation antidepressants such as SSRIs and SNRIs [26].

In the current study, the most common symptom for the referral was a suicidal risk at about 97.7%, psychotic symptoms at 96.9%, harm to others at 96.2%, and lastly poor response to treatment at 94.6%. Similar components for referral to psychiatrists were observed in the South African Family Medicine practice [10]. In contrast to the present study findings, a study conducted among tertiary care settings revealed that about 56.6% of patients were referred due to suicidal risk and 91.6% of patients were referred due to substance abuse [28]. This difference in prevalence could be due to study setting differences.

Regarding the challenging diagnosis of depression in elderly patients, approximately 76.2% of physicians emphasized the necessity for additional training related to geriatric depression. In a study involving family and general practitioners, 65.8% of the sample highlighted the importance of training to improve depression detection among the geriatric population. Furthermore, 56.6% of physicians mentioned that mental health conferences contribute to enhancing depression detection [13]. Another study also supported that training of various lengths for PHC physicians enhances confidence in the diagnosis of geriatric depression [29].

There was no statistically significant association observed between gender and other barriers to diagnosis and treatment aspects such as the elderly don’t accept questions in the study group. Almost the same finding of elderly people don’t accept questions for the geriatric diagnosis as a barrier observed in the Malaysian study [30].

In the present study, about 70% of the physicians gave a response as there are no MOH/QHC guidelines for the diagnosis and treatment of depression in the geriatric population. A study conducted in Bahrain also stated that 86.4% of physicians gave an opinion on having specific geriatric guidelines for the detection and management of depression [7].

Limitations of the study are that our questionnaire is a self-administered questionnaire, there is some misunderstanding of questions, and physicians' less responsive nature also made it difficult situation to complete the study, due to their busy schedules. The collection of data from a large number of PHCCs is the strength of the study.

## Conclusions

According to the study findings, four-fifths (85.4%) of PCPs diagnose depression, and on average every PCP sees two geriatric age group depressed patients per month. In their regular practice for the detection of geriatric depression, seven out of 10 (70%) physicians used the screening tool PHQ-2, followed by more than 53.8% of physicians using the geriatric depression scale. Physicians' most commonly triggered symptoms to screen depression for their patients were sad mood (94.6%) and loss of interest (92.3%) in more than nine out of 10 cases. More than one-fourth (26.2%) of the physicians did not use any lab tests for the depression diagnosis before the start of management. Nearly more than seven out of 10 (76.2%) physicians preferred non-pharmacological treatment for the initial plan of geriatric depression treatment. About the class of medication, more than seven out of 10 (73.1%) physicians preferred the SSRI class. Among SSRIs medications, escitalopram (46.9%) is prescribed most commonly by physicians. Regarding the barriers to diagnosing late-life depression, more than seven out of 10 physicians felt that they needed more training (76.2%) and short consultation time (70%). Study results denoted that the need to enhance awareness about guidelines at PHCC required regular and periodical training to be arranged for the physicians to increase the detection and management of depression among elderly people to provide a good quality of life.
